# Perceived factors that influence adoption, implementation and sustainability of an evidence-based intervention promoting healthful eating and physical activity in childcare centers in an urban area in the United States serving children from low-income, racially/ethnically diverse families

**DOI:** 10.3389/frhs.2022.980827

**Published:** 2022-11-04

**Authors:** Leilah Siegel, Yuka Asada, Shuhao Lin, Marian L. Fitzgibbon, Angela Kong

**Affiliations:** ^1^4-H Youth Development, University of Illinois Extension, St. Charles, IL, United States; ^2^Community Health Sciences, School of Public Health, University of Illinois Chicago, Chicago, IL, United States; ^3^Department of Kinesiology and Nutrition, University of Illinois at Chicago, Chicago, IL, United States; ^4^Institute for Health Research and Policy, University of Illinois Chicago, Chicago, IL, United States; ^5^Department of Pharmacy Systems, Outcomes and Policy, University of Illinois Chicago, College of Pharmacy, Chicago, IL, United States

**Keywords:** implementation, preschool, nutrition, adoption, evidence-based, intervention, sustainability

## Abstract

**Introduction:**

Early childcare centers offer optimal settings to provide healthy built environments where preschool age children spend a majority of their week. Many evidence-based interventions (EBIs) promoting healthful eating and physical activity for early childcare settings exist, but there is a limited understanding of how best to support adoption, implementation and sustainability in community settings. This study examined how early childcare teachers and administrators from Chicago-area childcare centers serving children from low-income, racially/ethnically diverse communities viewed an EBI called Hip to Health (H^3^), and the factors they perceived as relevant for EBI adoption, implementation, and sustainability.

**Methods:**

A multiple methods study including key informant interviews and a brief survey was conducted. Key informant interviews with teachers and administrators from childcare centers located in Chicago, IL were completed between December 2020 and May 2021. An interview guide and coding guide based on the Consolidated Framework for Implementation Research (CFIR) was developed. Interview transcripts were team coded in MAXQDA Qualitative Data Analysis software. Thematic analysis was used to identify findings specific to adoption, implementation, and sustainability. Participants were also asked to respond to survey measures about the acceptability, feasibility, and appropriateness of H^3^.

**Results:**

Overall, teachers (*n* = 20) and administrators (*n* = 16) agreed that H^3^ was acceptable, appropriate, and feasible. Low start-up costs, ease-of-use, adaptability, trialability, compatibility, and leadership engagement were important to EBI adoption. Timely and flexible training was critical to implementation. Participants noted sustainability was tied to low ongoing costs, access to ongoing support, and positive observable benefits for children and positive feedback from parents.

**Conclusions:**

These findings suggest that EBIs suitable for adoption, implementation, and sustainment in childcare centers serving racially/ethnically diverse, low-income families should be adaptable, easy to use, and low-cost (initial and ongoing). There is also some evidence from these findings of the heterogeneity that exists among childcare centers serving low-income families in that smaller, less resourced centers are often less aware of EBIs, and the preparation needed to implement EBIs. Future research should examine how to better support EBI dissemination and implementation to these settings.

## Introduction

Physical inactivity and poor diet quality are major drivers of chronic disease. Intervening early on in life to encourage children's engagement in physical activity and eating healthful foods may protect against non-communicable disease development (1–3). This is particularly important for low-income and racial/ethnic minoritized groups who are disproportionately impacted by chronic disease (4, 5). To address these health disparities and promote greater health equity, prevention efforts that promote healthful eating and physical activity need to have sufficient reach and be disseminated equitably.

Evidence-based interventions (EBIs) promoting physical activity and healthful eating that target preschool children in childcare settings have been shown to be effective in the United States and other high-income countries (6, 7). Embedding EBIs as part of standard programming in existing childcare settings has the potential to expand the reach of EBIs and improve children's health on a population basis. However, in practice, EBIs are not always readily adopted (8) and even if adopted, challenges to implementation and sustainability exist (8–10). A recent systematic review suggests that strategies to support EBI implementation are usually needed, but selection of strategies are largely dependent on the local context (9). Improving EBI translation to real world practice settings requires a greater understanding of why and how childcare centers implement these types of programs. It also requires a greater understanding of the factors needed for such EBIs to be successfully sustained.

This study assessed how teachers and administrators in an urban area in the United States viewed a specific EBI. Hip Hop to Health (H^3^) is an EBI that was developed for and previously tested in Head Start classrooms for African American and Latinx preschool children to be delivered by preschool teachers (11, 12). This study examined the factors that teachers and administrators perceived as relevant to this EBI's adoption, implementation, and sustainability in childcare settings serving children from low-income and/or racial/ethnic minoritized families.

## Methods

### Hip hop to health (H^3^) EBI

H^3^ is an EBI that was developed to be delivered by teachers in childcare settings serving African American and Latinx children. The randomized effectiveness trial testing H^3^ found significant between-group differences in physical activity, screen time, and diet quality that favored the intervention group (11, 12). H^3^ consists of eight lessons which feature activities on topics such as “Go and Grow Foods vs. Slow Foods,” “Grains,” “Vegetables,” and “Drinking Water and Moving Your Body.” “Go and Grow Foods” are healthy foods that should be eaten often, whereas “Slow” foods are foods that should be eaten in moderation. Each lesson is 35–40 minutes and consists of 20 mins of physical activity that can be done along with an accompanying musical soundtrack. Lessons also include additional activities such as reading stories, sampling foods, and using puppets (11).

### Study design

This is a multiple methods study including a survey and key informant interviews. Both qualitative and quantitative methods were used to obtain a more complete picture of the implementation, adoption, and sustainability factors. Data were gathered from early childcare administrators (*n* = 16) and teachers (*n* = 20) between December 2020 and May 2021. The study was reviewed and approved by the Institutional Review Board at the University of Illinois Chicago (protocol # 2020-0139) which reviewed the ethics and protection of the rights and welfare of the individuals involved in the proposed research.

### Study approach

Participants were verbally consented and then asked to watch a brief video describing the H^3^ curriculum. Following the video, participants were shown a sample lesson from H^3^ and completed a brief survey collecting quantitative data. Qualitative data was then collected *via* a semi-structured interview by a trained staff member.

### Sampling, setting, and key informants

Chicago is the third largest city in the United States and has a population of over 3 million people (13). Demographically, Chicago's population is nearly evenly split between white residents (33%), African American residents (29%), and Latinx residents (29%) (13). Over 20% of city inhabitants live below the national poverty line, with this rate varying based on race and ethnicity: 32% of African American residents and 22% of Latinx residents live below the poverty line as compared with 10% of white residents (13).

Forty-five childcare centers were initially identified based on purposive sampling of those (1) located in Chicago or surrounding suburbs; (2) serving a population of 3–5 years of age; and (3) serving a largely low-income or African American or Latinx population. Head Start program participation was noted but not required.

The childcare centers were purposively sampled from (a) a list of Head Start centers that the senior author had generated from a previous study, and (b) online searches of early childhood centers in Chicago or surrounding suburbs that met additional criteria described above. Some sites were no longer active or were not able to be reached.

Teachers and administrators from these centers were identified *via* contact information online, or from the list of contacts of Head Start centers. An email describing the study was sent to these teachers and administrators inviting them to participate in the study. The email contained an attached flyer describing the study in more detail as well as the informed consent document. Teachers and administrators from 22 different centers indicated interest and were scheduled for interviews. Interview materials were only available in English so self-reported comfort with speaking and reading English was a requirement for participation.

Teacher interviews were conducted with early childhood education staff whose job titles were teacher, teacher's aide, or instructional coach. Teachers were interviewed because of their key role in EBI implementation; their perceptions and receptivity to the EBI are critical components to implementation. Administrator interviews were conducted with site directors, coordinators, nutritionists, and education managers at early childcare centers. They represent center leadership and are often involved in decision making; their insights are particularly important for learning about organizational support and capacity of the center.

### Interview procedures

All interviews were conducted *via* Zoom by a trained staff member in English. The staff member conducting the interviews identifies as African American, with the majority of the research team identifying as persons of color (Asian/Asian American). Previous studies have cited that mistrust, implicit bias, and lack of cultural competence could serve as barriers to individuals from racially minoritized backgrounds participating in research; these individuals are more likely to participate when they perceive that the researcher is similar in background to themselves (14–17).

A semi-structured interview guide was developed for the key informant interviews, guided by the Consolidated Framework for Implementation Research (CFIR) (18). CFIR is a meta-theoretical framework that can be used to identify barriers and facilitators related to EBI adoption, implementation, and sustainability. The interview guide included select CFIR constructs within the following domains: (1) intervention characteristics, (2) outer setting, (3) inner setting, and (4) characteristics of the individual. A summary of constructs included for each domain is summarized in the Supplementary Table. Key informants were asked to provide their input about H^3^. The questions also captured demographic information and key informants' previous experiences with adopting, implementing, and sustaining similar programs. Before finalizing the interview guide, pilot interviews were conducted with five teachers and administrators from the target population to check for clarity, correct terminology, and flow. The interview guide was revised several times following pilot interviews. Slight variations in the questions used in the interview guides were also included to make the questions more relevant for a teacher or an administrator. Verbal informed consent was obtained from each participant before beginning the interviews. All interviews were audio recorded and professionally transcribed.

### Survey procedures

Acceptability, feasibility, and appropriateness of the EBI (i.e., H^3^) were assessed using the Acceptability of Intervention Measure (AIM), Feasibility of Intervention Measure (FIM), and Intervention Appropriateness Measure (IAM) developed by Weiner et al. (19). Each measure has four items assessed on a five-point Likert scale with responses ranging from completely disagree to completely agree; higher scores reflect better acceptability, feasibility, and appropriateness. Participants were asked to respond to survey questions after watching the brief video and before beginning the qualitative interview. All participants completed both the qualitative interview and the quantitative survey.

### Data analysis and management

#### Qualitative analysis

An *a priori* draft codebook was created following the CFIR-informed interview guide and revised during several rounds of coding. All transcripts were uploaded into MAXQDA Qualitative Analysis software (20). To begin, coders (LS, YA, SL, AK) independently coded a subset of the transcripts to discuss discrepancies in coding and revise coding definitions as needed. Coders then met weekly to discuss coding progress, further refine the coding guide, and to identify patterns. Once coders reached approximately >85% inter-rater agreement and no additional revisions were required of the coding guide, each remaining transcript was double coded. Coders used MAXQDA functions such as code matrices and summary grids to visualize data and look for cross-cutting patterns. Based on weekly team discussions and iterative revisions to data displays, themes specific to adoption, implementation, and sustainability were developed and documented (21).

#### Quantitative analysis

Descriptive statistics, presented as means or percentages as appropriate, were calculated to describe the study sample and to summarize FIM, AIM, and IAM scores.

## Results

### Demographic characteristics

Table 1 describes characteristics of key informants and the childcare centers where they are employed. Twenty early childcare teachers and 16 administrators were interviewed; most were affiliated with Head Start programs (95% of teachers, 62% of administrators), with the majority of teachers and administrators holding their positions for more than 6 years. Interview respondents were all female; 56% of administrators and 40% of teachers self-identified as African American, and 6% of administrators and 21% of teachers self-identified as Hispanic. The highest level of education obtained for most administrators was a master's degree (50%); the highest level of education for the majority of teachers was a bachelor's degree (55%). The average age of all respondents was 47.5 years. Fifty percent of administrators and 70% of teachers practiced in *large* centers, defined as centers with multiple locations serving more than 100 children. The remaining practiced in single site settings. *Mid-size* was defined as single site centers with more than 50 children and *small* was defined as single site centers with <50 children.

**Table 1:** Interview participant and childcare setting characteristics.

**Demographics**	**Administrators (*n* = 16)**	**Teachers (*n* = 20)**
**Sex (female)**	100%	100%
**Mean age in years**	48	47
**Ethnicity**		
Hispanic	6%	21%
Non-Hispanic	94%	73%
**Race**		
African American	56%	40%
Native American	0%	5%
Two or more races	6%	5%
White	38%	50%
**# of years in position**		
0–5	19%	20%
6–10	31%	40%
11–20	31%	20%
21+	19%	20%
**Highest level of education completed**		
High school diploma	0%	10%
Associate's degree	12%	15%
Bachelor's degree	38%	55%
Master's degree	50%	20%
**Center location**		
Suburban	69%	50%
Urban	31%	50%
**Head start?**		
N	38%	5%
Y	62%	95%
**Center size**		
Small (1 site < 50 students)	44%	5%
Mid-size (1 site > 50 students)	6%	25%
Large (Multiple sites < 100 students)	50%	70%

### Quantitative data results: Acceptability, feasibility, and appropriateness of the EBI

Participants provided their impressions of H^3^'s acceptability, feasibility, and appropriateness by responding to AIM, FIM, and IAM survey items. Table 2 reports AIM, IAM, and FIM mean scores by individual item and category totals. Most teachers and administrators agreed that H^3^ was acceptable (mean: 4.24, SD: 0.50), feasible (mean: 4.31, SD: 0.46), and appropriate (mean: 4.21, SD: 0.47).

**Table 2:** Mean scores for AIM, FIM, IAM (individual and total).

**Item**	**Description**	**Mean (SD)**	**Range**
AIM: Approval	Hip Hop to Health meets my approval	4.08 (0.84)	1–5
AIM: Appeal	Hip Hop to Health is appealing to me	4.39 (0.60)	3–5
AIM: Welcome	I welcome Hip Hop to Health	4.31 (0.58)	3–5
AIM: Like	I like Hip Hop to Health	4.19 (0.58)	3–5
	**Total**	**4.24 (0.50)**	
FIM: Implement	Hip Hop to Health seems implementable	4.11 (0.54)	3–5
FIM: Possible	Hip Hop to Health seems possible	4.36 (0.54)	3–5
FIM: Doable	Hip Hop to Health seems doable	4.39 (0.49)	4–5
FIM: Easy	Hip Hop to Health seems easy to use	4.28 (0.61)	
	**Total**	**4.31 (0.46)**	
IAM: Fitting	Hip Hop to Health seems fitting	4.28 (0.57)	3–5
IAM: Suitable	Hip Hop to Health seems suitable	4.19 (0.47)	3–5
IAM: Applicable	Hip Hop to Health seems applicable	4.19 (0.58)	3–5
IAM: Match	Hip Hop to Health seems like a good match	4.17 (0.61)	3–5
	**Total**	**4.21 (0.47)**	

AIM, Acceptability of the Intervention Measure; FIM, Feasibility of the Intervention Measure; IAM, Intervention Appropriateness Measure.

Teachers (n = 20) and Administrators (n = 16).

#### Qualitative data results: Factors influencing adoption, implementation, and sustainability

Table 3 summarizes main themes that emerged from the key informant interviews and are organized by CFIR domains and constructs along with accompanying quotes. Most themes were related to CFIR constructs within the domains of “*intervention characteristics*” (i.e., cost, adaptability, trialability, complexity) and “*inner setting*” (i.e., compatibility, available resources, leadership engagement) (12). Themes cut across adoption, implementation, and sustainability as shown in Table 3 and are described further in the next sections.

**Table 3:** Adoption (A), implementation (I), and sustainability (S) of a nutrition and physical activity evidence-based intervention (EBI) in childcare centers: main themes from key informant interviews.

**CFIR Domains:**	**A**	**I**	**S**	**Example quotes**
**Constructs**				
***Themes***				
**Intervention Characteristics: Costs**	x	x	x	“I mean, definitely cost because we are funded by grants, so whatever dollar amount is allocated for health and nutrition, education would definitely play a role in implementing the curriculum.” (Teacher ID 2010)
*Initial and ongoing costs are reasonable*				“Cost. That's the big one.” (Administrator ID 1005)
**Intervention Characteristics: Adaptation**	x	x	x	“That's the part that I was wondering about is with the flexibility and I feel like if the 20 mins are divided, that it could be done...break up program into smaller chunks.” (Adminstrator ID 1004)
*Can be adapted to fit into current practices and routines*				“Maybe having two kits per classrooms in case you want to do it in smaller groups. Instead of a classroom of 20 trying to do it, you know, maybe two teachers are doing it at different times.” (Administrator ID 1002)
				“I mean, because I don't know it thoroughly, I guess I would say maybe the language part, maybe just adding different languages in there. Maybe not for kids so much but for families. Cause we do flyers home and, you know, I think they would appreciate it a lot more if they were able to read it and understand it. So maybe that would be a good to change.” (Teacher ID 2011)
**Intervention Characteristics: Trialability**	x			“… obviously learning about it and then maybe having the opportunity to try it, like pilot it in a couple of classrooms and then you know, see how it goes and then make the decision as to purchase it for the entire program.” (Administrator ID 1009)
*Ability to pilot the program and to gather feedback from teachers*				“She would definitely want the teachers to try it out and then get feedback because she does trust teacher input, you know, after her own evaluation, see if it, she thinks it would be successful in the classroom and then give it that test run and then ask for feedback from the teachers on whether or not it was successful or what could make it successful.” (Teacher ID 2012)
**Intervention Characteristics: Complexity**	x	x	x	“Again, it just really boils down to it being easy, not something that's a burden on the teachers feeling like it's another task to do in the classroom.” (Administrator ID 1008)
*Curriculum must be easy to use*				“And then I would look at how difficult, or how simple it is to, to put together or to, to implement the curriculum. And then I would look at how much work, how much additional work and how many additional supplies would be needed. That's normally what has either caused me to use a curriculum or to let it fall off.” (Administrator ID 1016)
**Inner Setting: Compatibility**	x	x	x	“…we already use food experiences and exercise and stuff like that. I think it would just be ongoing because it'll be a part of our curriculum. That's something we already do anyway.” (Teacher ID 2005)
*Aligns with priorities, standards, and current practices*				“Well, I think it should make sure it fits into the Head Start standards for sure. And other, the PI or the PFA standards. And to just make sure that there's no, I mean, I don't think there's anything that is culturally inappropriate. That it can fit into the program day. I mean, I kind of don't know, but those are things that I imagine are important.” (Administrator ID 1004)
**Inner Setting: Available Resources:** Training is critical to implementation and maintenance. Training needs to be timely, flexible, and ongoing.	x	x	x	“Would definitely require constant training to keep it going. Cause we, you know, often in programs we start things, we stop, we start, stop.” (Administrator ID 1002)
				“The long-term cost availability continued trainings and support…” (Administrator ID 1010)
				“It would be accepted if it's presented and implemented in a timely fashion. If this were to be something that we were to pilot, it would need to start in August, educate the staff so they can learn it and then bring it in. When the children come in, if it were to be something that was brought in in November or December, it would be more difficult because since we're grant funded, we have several deadlines. So to be most effective, it seems that it's, that a timeline would be implemented in the August early August, teaching the teachers and then them full speed ahead at end of August when the children were to come on, would be the most effective way. It's crucial the timeline, the one you would present it to teachers to be very honest.” (Administrator 1005)
**Inner Setting: Leadership Engagement**	x	x	x	“it's a matter of getting them [administrators] on board and then also have a creating that time for, for training the teachers and getting the resources. Important to leadership on board and providing training and resources to support teachers in implementation” (Teacher ID 2012)
*Commitment from administrators and others with decision making capabilities*				
				“Yes, the parents I mean, in the office of Head Start there's a parent board. So they have also with budgets, they have to get it approved by the parent board.” (Teacher ID 2004)
**Positive benefits to children**			x	“Well, because if it proves to be beneficial, it benefits the program. I guess there's some more success stories about, you know, the overall wellbeing of families and children” (Teacher ID 2009)
*Maintenance of the EBI is strengthened if there is evidence that children are benefiting from the program*.				“Long-Term? I think again, I think the child's outcomes, so if children are, retaining the information and the curriculum is successful in getting that, I think that we would not have a reason to change.” (Administrator ID 1009)

##### Adoption

Understanding factors that lead to the adoption of an EBI helps researchers to both adapt and design future interventions. Constructs particularly relevant to EBI adoption were cost, adaptability, trialability, complexity (i.e., ease of use), compatibility, and leadership engagement.

##### Costs: Initial costs are reasonable

CFIR defines the construct of “cost” as “costs of the intervention and costs associated with implementing the intervention, including investment, supply, and opportunity costs” (18). Initial or start-up costs were of particular importance in considering whether to adopt a curriculum. Interview participants were given a sample one-time curriculum price of $65 and asked if they thought that cost was “reasonable.” Many participants from *large* centers stated that the cost was reasonable since they'd “*paid far more”* for other curricula materials. Many participants from *small* centers reported that the amount quoted was reasonable because it was lower than the amount they had in mind, even though they did not have other curriculum to compare it to.

##### Adaptability: Can be adapted to fit into current practices and routines

Adaptability is defined as, “the degree to which an intervention can be adapted, tailored, refined, or reinvented to meet local needs” (18). Both teachers and administrators described intervention adaptability as important to its adoption. Specifically, they mentioned several characteristics of adaptability, such as (1) having the intervention translated into multiple languages; (2) being able to modify the length or use it as a series of separate modules; (3) adapting it for slightly younger or older children; and (4) being able to modify it to for virtual use (Table 3).

##### Trialability: Ability to pilot the program and to gather feedback from teachers

Trialability is a CFIR construct that is defined as: “The ability to test the intervention on a small scale in the organization, and to be able to reverse course (undo implementation) if warranted.” (18). Teachers and administrators mentioned that being able to pilot the curriculum before deciding whether or not to adopt it was very important. Additionally, it would be important to obtain positive feedback from teachers before committing to a program (Table 3).

##### Complexity: Curriculum must be easy to use

Complexity is defined as “perceived difficulty of the intervention, reflected by duration, scope, radicalness, disruptiveness, centrality, and intricacy and number of steps required to implement” (18). Many administrators and teachers expressed the need for the EBI to be easy to use, which considered multiple dimensions. One administrator commented that adopting the curriculum “*should not [be] something that's a burden on the teachers…like it's another task to do in the classroom*.” Curriculum that is disruptive to current workflow and practices or had too many steps would be barriers to adoption (Table 3).

##### Compatibility: Aligns with priorities, standards, and current practices

Compatibility is defined as “the degree of tangible fit between meaning and values attached to the intervention by involved individuals, how those align with individuals' own norms, values, and perceived risks and needs, and how the intervention fits with existing workflows and systems” (18). Teachers and administrators responded that it was important that an intervention fit their organization's values, norms, and policies to be considered. Many participants reported that their organizations placed a priority on student health and that they already implemented activities to promote nutrition and/or physical activity. For teachers and administrators affiliated with Head Start, an EBI that aligned with Head Start standards was of great importance for adoption.

##### Leadership engagement: Commitment from administrators and those with decision making capabilities

CFIR describes leadership engagement as “commitment, involvement, and accountability of leaders and managers with the implementation” (18). Engagement from leadership is of particular importance in larger centers such as the Head Start affiliated centers. Buy-in from administrators is necessary to support organizational capacity at all phases, but it is of particular importance when deciding to adopt a program. One teacher mentioned “*it's a matter of getting them [administrators] on board and then also creating that time for training the teachers and getting the resources*.” Centers affiliated with Head Start had to get approval from different boards before programs were adopted, including an advisory board consisting of parents (Table 3).

#### Implementation

Many of the constructs relevant to adoption are also relevant to EBI implementation as summarized in Table 3. However, there was a particular emphasis on training (CFIR construct: Available resources) to support EBI implementation.

##### Available resources: Training needs to be timely and flexible

The construct “Available Resources” is defined as: “the level of resources dedicated for implementation and on-going operations, including money, training, education, physical space, and time” (18). Both teachers and administrators described training as being critical for implementation; specifically, two main considerations included: (a) timing; (b) delivery/format. First, trainings should be offered when teachers were onboarded for the school year and received trainings for other curriculum/procedures (e.g., in August). This was particularly relevant for Head Start centers.

Second, when teachers were asked about optimal training delivery, many saw advantages to both online offerings and in person. Many expressed a preference for hands-on learning, but they also liked the convenience and permanence of online trainings.

#### Sustainability

Interview respondents were asked if they could see their center using the H^3^ curriculum in the long term, and what factors would influence their center's ability to use the curriculum in the long term. The most common themes related to EBI sustainability were ongoing costs, training support, and evidence that children are positively benefiting from the program.

##### Cost: Ongoing costs need to be manageable and to support ongoing training

Both teachers and administrators stated that low on-going costs were extremely important in sustaining EBI implementation. Specific costs mentioned were for printing, fuel, food, and replacing program components.

Ongoing support or continued training was also mentioned as a crucial factor for EBI sustainability. In large centers in particular, there is a consistent need to offer training to current staff in the form of booster sessions and to train new teachers since staff turnover is common.

##### Positive benefits to children: Need to see evidence that the EBI is working

Both teachers and administrators reported the importance of seeing children's positive reactions and benefits from the program in observable ways. In addition, many teachers would consider the program successful if the benefits also extended to parents.

#### Differences by center size and type

In general, larger or multi-site centers, such as Head Start centers, had adopted EBI programs in the past. Participants from these centers reported more familiarity and readiness, as well as cited existing regulations and policies that support nutrition and physical activity curricula. One administrator stated: “*Some months we have a focus, it could be portion sizing, it could be a healthy eating activity. And we do this program once a year; they come in and they teach the kids. It's not the teacher's doing it. It's this organization doing it. And then the children get to take something home with them, for example, like the plates, little dividing of the plate for the serving sizes. So that's currently what we do*.” In contrast, none of the participants from *small* centers in this study had implemented a formal EBI previously. Many expressed “creating” their own program by pulling together resources or using those that were given to them. One teacher said: “*Our director, every week she sends recipes about healthy nutrition so we can show [them] to the kids and share [them] with the parents. Every week we do that. And for the physical activity, other than going to the playground in the shade or simple activities in the classroom, like dancing, that's it. That's all*.”

## Discussion

These findings highlight factors related to EBI adoption, implementation, and sustainability in childcare centers within an urban area in the United States serving low-income, racially/ethnically diverse families. Successfully adopting, implementing, and sustaining an EBI promoting positive health behaviors in early childhood can be one strategy to promote greater health equity in these populations. In this study, teachers and administrators responded favorably to the EBI (i.e., H^3^) presented to them and agreed that it was acceptable, appropriate, and feasible. In considering EBI adoption, implementation, and sustainability, respondents stressed the need for the EBI to fit into what they were already doing. It also needed to be low-cost (start-up, ongoing), easy to use, and have training supports that were flexible to the needs of the center and would be ongoing. However, there were notable differences between small and large centers in their readiness and capacity for EBI adoption that warrant further attention.

Teachers and administrators interviewed were largely in favor of the EBI proposed as reflected in both qualitative and quantitative findings (e.g., AIM, FIM, IAM scores) (19). In most cases, centers were already promoting physical activity and healthful eating in some form; therefore, many viewed the EBI as compatible with existing practices and could reinforce what they were already doing. Compatibility has been recognized as a facilitator to program adoption based on previous studies of physical activity and nutrition interventions delivered in childcare settings (22–30). For example, EBIs that “fit well within existing curricula”), “enhanced the classroom,” or were aligned with existing “preschool and government health objectives” were considered facilitators to implementation (23, 24, 27).

There was also consensus among teachers and administrators that EBIs needed to be easy to use and could be easily adapted to a center's routine or practices. The adaptations most often mentioned by key informants in the current study included breaking up sessions into shorter lessons to accommodate daily routines and adapting lesson plans to accommodate varying class sizes, age groups, and language needs (e.g., translation of parent handouts). Similar adaptations have been identified in previous studies. These studies included settings with predominantly white populations (e.g., Sweden and Scotland); however, the income status of families with children enrolled in the centers was not reported (23, 27, 31, 32) as is often the case in many of these studies. The theme of adaptability was also found in studies conducted in Head Start centers which serve low-income families (26, 33, 34), which is more similar to the target population in our study. Implementation also occurs more smoothly when interventions are perceived as easy to use, require little to no preparation (e.g., ready to use), and are not overly burdensome. This facilitator to implementation (ease of use) has been largely reported in centers with predominantly white populations (income status not reported) (23, 24, 31, 35, 36). When this theme (ease of use) was reported in racially/ethnically diverse settings and/or Head Start centers (22, 25, 37), it was more common to perceive interventions in terms of its complexity rather than ease of use. For instance, interventions viewed as too complex and therefore difficult to implement were those with too many activities, had excessive paperwork, or required too much planning. In contrast to our findings, these themes were gathered after an intervention was implemented and therefore, centers could speak better to the challenges they encountered with implementation.

Cost was important to both EBI adoption and sustainability. Both administrators and teachers mentioned that the cost of the intervention was an essential factor for deciding to use the curriculum and being able to continue to use it over time. Specific cost-factors that were mentioned were the initial cost of the curriculum, ongoing costs such as replacing materials that became lost or worn out, and food costs. This highlighted the importance of considering the cost to maintain the curriculum over time (beyond startup costs). This finding was addressed by Eismann et al. (38), who reported that organizations often fail to successfully implement EBIs in part because they do not realize up front what costs will be needed to sustain the intervention. Burton et al. (39) noted that participants perceived the cost of their EBI (including “investment, supply, and opportunity cost”) was prohibitive and a barrier to implementation. Consideration of cost and cost effectiveness is not often reported in studies examining healthy eating and physical activity practices or programs in childcare settings as noted by previous systematic reviews on physical activity and healthy eating interventions in childcare settings (40, 41).

Another key finding was the importance that both administrators and teachers placed on being well-trained to implement the intervention, which is critical for the success of any EBI. This was consistently found in studies across contexts including centers serving populations that were predominantly white, racially/ethnically diverse, and low-income (22–25, 27, 30, 32–34, 36, 42). As emphasized in this current study, trainings should be planned with the partner organizations to adequately consider their needs and preferences. Specifically, the type of training, whether online, in-person or a hybrid, as well as the timeline of training were mentioned as being critically important. Due to the calendar of the school year, having trainings begin shortly after teachers arrive back at school in August was mentioned as a key factor to implementation success. Also, the availability of resources, ongoing trainings and support from the university, and a designated contact person that teachers and administrators can contact for help or with questions were listed as being extremely important for sustaining an EBI. A 2021 paper by Combs et al. reported that training is a key part of EBI implementation, but that training must be conducted in a manner that is most useful to the center in terms of scheduling and mode (43). Teachers in the current study reported both pros and cons to online training—while it offered additional convenience it lacked a hands-on component that many early childhood teachers reported was very helpful when learning a new curriculum. Combs et al. also found that while online training did not lead to lower levels of adherence to the curriculum or dosage, it was associated with lower reports of quality of delivery (43). Their recommendation was to include some experiential component to online training; the findings of this paper support that recommendation.

Finally, it is important to note some differences by center type/size that could have implications for EBI adoption and dissemination. In general, centers affiliated with Head Start were larger and/or part of multi-site centers. When interviewing administrators and teachers from Head Start centers, most had implemented EBIs or similar programs in the past so there were mechanisms in place and organizational capacity to support EBI adoption and implementation. In contrast, key informants from small, single site centers were not at all familiar with EBIs; however, they too, prioritized promoting physical activity and healthful eating in their centers. This suggests that EBI dissemination may favor centers that are larger, have greater organizational capacity (e.g., leadership, available resources, etc.), and are more likely to be networked with external organizations (e.g., academic institutions). Researchers have a role in perpetuating this bias since the development and testing of EBIs usually originate from grant funded studies carried out by academic institutions. A gap in the current research is the equitable dissemination of EBIs (44). Addressing this gap means better dissemination of EBIs through potential systems and policy changes, as well as developing implementation strategies to support EBI adoption and implementation in smaller, less resourced and networked centers.

### Limitations

These findings have some limitations. This study used purposive sampling methods that recruited teachers and administrators from childcare centers based on characteristics (e.g., centers serving low-income, racially/ethnically diverse families) that were representative of the target population of this study and could speak to the phenomenon under investigation. One limitation of purposive sampling is that it can be prone to researcher bias, since the researcher is making a decision about who to sample (45). Another possible limitation is that these results may not be representative of EBI facilitators and barriers outside of the studied population (46). However, this approach was still used as it provided the most time and resource-effective means of recruiting the targeted population due to challenges during the COVID-19 pandemic. A purposive sampling approach also provided additional insight into EBI implementation within this specific population.

This was also a cross sectional study that captured perceived views, thoughts, and insights from key informants at one point in time, prior to EBI implementation. Cross sectional designs are limited in their ability to deduce a causal relationship between the variables being studied and to describe a phenomenon over a period of time (47). However this design allowed for a relatively timely and straightforward study. A cross sectional design also allowed for the study of multiple possible implementation factors concurrently (47). Lastly, a third possible limitation is the relatively prospective nature of these findings; however, assessing the EBI prior to implementation may save valuable time and resources when H3 is fully implemented, and ultimately lead to a more impactful intervention.

## Conclusions

Overall, the study findings indicate that EBIs should be easy to implement, low-cost (initial and ongoing), have proper training supports, and be compatible with the practices and policies of early childcare centers to be successfully adopted, implemented, and sustained. Further attention should also be given to more equitable dissemination of EBIs and understanding how to support the adoption, implementation, and sustainability of EBIs in smaller, less-resourced centers.

## Data availability statement

The raw data supporting the conclusions of this article will be made available by the authors, without undue reservation.

## Ethics statement

The studies involving human participants were reviewed and approved by Institutional Review Board of the University of Illinois Chicago (Protocol # 2020-0139). Written informed consent for participation was not required for this study in accordance with the national legislation and the institutional requirements.

## Author contributions

AK, LS, MF, and YA contributed to the design of the study. LS collected the data and wrote the first draft of the manuscript. YA directed the qualitative analysis. LS, YA, AK, and SL analyzed and interpreted data. LS and AK developed the figures and tables. All authors reviewed the manuscript, provided critical feedback, and approved the final manuscript.

## Funding

Research reported in this publication was supported in part by the University of Illinois Cancer Center Pilot Project Program funds.

## Conflict of interest

The authors declare that the research was conducted in the absence of any commercial or financial relationships that could be construed as a potential conflict of interest.

## Publisher's note

All claims expressed in this article are solely those of the authors and do not necessarily represent those of their affiliated organizations, or those of the publisher, the editors and the reviewers. Any product that may be evaluated in this article, or claim that may be made by its manufacturer, is not guaranteed or endorsed by the publisher.

## Abbreviations

EBI: Evidence based intervention
CFIR: Consolidated Framework for Implementation Research
H^3^: Hip Hop to Health
AIM: Acceptability of Intervention Measure
FIM: Feasibility of Intervention Measures
IAM: Intervention Appropriateness Measure
SD: Standard deviation.
