# Supplementary material for: Perceived factors that influence adoption, implementation and sustainability of an evidence-based intervention promoting healthful eating and physical activity in childcare centers in an urban area in the United States serving children from low-income, racially/ethnically diverse families
Source: Front Health Serv. 2022 Nov 4;2:980827. doi: 10.3389/frhs.2022.980827 (PMC10012626; doi:10.3389/frhs.2022.980827)
Supplement: Supplementary file 1 [file Table_1.DOCX]

| **Supplemental Table.** CFIR Domains, Constructs, and Sample Interview Guide Questions | | | |
| --- | --- | --- | --- |
| Consolidated Framework for Implementation Research (CFIR) | | |  |
| Domains | Constructs | Definition | Sample questions from interview guide |
| Characteristics of the individual | Knowledge & Beliefs about the intervention | Individuals’ attitudes toward and value placed on the intervention as well as familiarity with facts, truths, and principles related to the intervention. | Based on what you’ve learned so far about the [curriculum], do you think it has the potential to improve eating and physical activity for students in your school/center? |
|  | Self-efficacy | Individual belief in their own capabilities to execute courses of action to achieve implementation goals. | Do you feel confident that you can implement the nutrition education aspects of the H^3^ curriculum? Yes or no. Please explain. |
| Intervention Characteristics | Source and Evidence Strength | Stakeholders’ perceptions of the quality and validity of evidence supporting the belief that the intervention will have desired outcomes. | How important is it to you that a [curriculum] adopted by your organization is based on research from a reputable source? |
|  | Complexity | Perceived difficulty of the intervention, reflected by duration, scope, radicalness, disruptiveness, centrality, and intricacy and number of steps required to implement. | What kind of challenges do you believe you may find with the H^3^ [curriculum]? |
|  | Cost | Costs of the intervention and costs associated with implementing the intervention including investment, supply, and opportunity costs. | For each new classroom that implements the H^3^ [curriculum] (bag, lessons, CD, etc.) there is a one-time, per classroom cost of about $65 once the study ends. Do you think that cost is reasonable? Yes or no. Please explain. |
|  | Relative Advantage | Stakeholders’ perception of the advantage of implementing the intervention versus an alternative solution. | How does the H^3^ [curriculum] compare to those other [curriculum]s or [curriculum](?) |
| Outer Setting | External policies and practices | A broad construct that includes external strategies to spread interventions, including policy and regulations (governmental or other central entity), external mandates, recommendations and guidelines, pay-for-performance, collaboratives, and public or benchmark reporting. | What kind of local, state, or national performance measures, policies, regulations, or guidelines will influence the success of implementing H^3^? |
|  | Cosmopolitanism | The degree to which an organization is networked with other external organizations. | Do you interact with groups or organizations that might be helpful for implementing the [curriculum] (e.g. school system, health clinics, after school, childcare, parks and recreation) in the community? If yes: Which group and organizations do you interact with? |
| Inner Setting | Compatibility | The degree of tangible fit between meaning and values attached to the intervention by involved individuals, how those align with individuals’ own norms, values, and perceived risks and needs, and how the intervention fits with existing workflows and systems. | How well does the H^3^ [curriculum] fit with the values and norms within your school/center?  How, if at all, would the H^3^ [curriculum] fit in with your existing workday? |
|  | Readiness for implementation | Tangible and immediate indicators of organizational commitment to its decision to implement an intervention. | What do you think will be the general level of receptivity from your organization to the H^3^ [curriculum]? |
|  | Adaptability | The degree to which an intervention can be adapted, tailored, refined, or reinvented to meet local needs. | What, if any, kinds of alterations do you think will be needed to make the H^3^ [curriculum] so that it will work effectively in your classroom |
|  | Available resources | The level of resources dedicated for implementation and on-going operations, including money, training, education, physical space, and time. | Do you expect to have sufficient resources to implement and administer the H^3^ [curriculum]? Yes or no. Please explain. |
|  | Leadership Engagement | Commitment, involvement, and accountability of leaders and managers with the implementation. | What is the process that person goes through to decide whether to use or keep a [curriculum] or not? |
|  | Organizational incentives & rewards | Extrinsic incentives such as goal-sharing awards, performance reviews, promotions, and raises in salary, and less tangible incentives such as increased stature or respect. | In the past, what incentives, special recognitions or rewards for teachers have helped with implementing a new [curriculum]? Please explain. |
|  | Networks and Communications | The nature and quality of webs of social networks and the nature and quality of formal and informal communications within an organization. | How is new information typically shared across the organization, such as new initiatives, accomplishments, issues, new staff, staff departures? |
|  | Relative priority | Individuals’ shared perception of the importance of the implementation within the organization. | If you decide to use H3, is there anything you can think of that would get in the way of implementing it at your center or organization? |
| Other (non-CFIR) | Parental support |  | What do you think would be necessary to increase parent engagement and awareness of the H^3^ [curriculum]? |
|  | Maintenance |  | What factors would influence your center’s ability to use this curriculum long-term? |
